# An ancient Turing-like patterning mechanism regulates skin denticle development in sharks

**DOI:** 10.1126/sciadv.aau5484

**Published:** 2018-11-07

**Authors:** Rory L. Cooper, Alexandre P. Thiery, Alexander G. Fletcher, Daniel J. Delbarre, Liam J. Rasch, Gareth J. Fraser

**Affiliations:** 1 Department of Animal and Plant Sciences, University of Sheffield, Sheffield, UK.; 2 School of Mathematics and Statistics, University of Sheffield, Sheffield, UK.; 3 Department of Earth Sciences, University of Oxford, Oxford, UK.; 4 Human Developmental Biology Resource, Institute of Child Health, University College, London, UK.; 5 Department of Biology, University of Florida, Gainesville, FL, USA.

## Abstract

Vertebrates have a vast array of epithelial appendages, including scales, feathers, and hair. The developmental patterning of these diverse structures can be theoretically explained by Alan Turing’s reaction-diffusion system. However, the role of this system in epithelial appendage patterning of early diverging lineages (compared to tetrapods), such as the cartilaginous fishes, is poorly understood. We investigate patterning of the unique tooth-like skin denticles of sharks, which closely relates to their hydrodynamic and protective functions. We demonstrate through simulation models that a Turing-like mechanism can explain shark denticle patterning and verify this system using gene expression analysis and gene pathway inhibition experiments. This mechanism bears remarkable similarity to avian feather patterning, suggesting deep homology of the system. We propose that a diverse range of vertebrate appendages, from shark denticles to avian feathers and mammalian hair, use this ancient and conserved system, with slight genetic modulation accounting for broad variations in patterning.

## Teaser

Diverse skin appendages, from shark denticles to bird feathers, develop via a conserved and ancient Turing patterning mechanism.

## INTRODUCTION

Vertebrates have a plethora of diverse epithelial appendages, including hair, feathers, scales, spines, and teeth (*1*). Recent research has revealed that these structures share extensive developmental homology, as they grow from a common foundation: the epithelial placode (*2*–*4*). Despite this shared ancestry, there are broad variations in both the final morphology and the spatial arrangement of these organs (*1*). Such variation in patterning has evolved to facilitate diverse functions, for example, drag reduction, thermoregulation, and communication (*5*–*7*).

Alan Turing’s reaction-diffusion (RD) model provides an explanation for the diversity of patterning observed in nature (*8*–*12*). This model describes how interactions between morphogens diffusing differentially through a tissue can give rise to autonomous patterning of epithelial appendages (*8*, *13*). These morphogens typically constitute two interactive molecular signals that occupy the role of a short-range activator and long-range inhibitor (*14*). The autocatalytic activator promotes its own expression and expression of the inhibitor, which, in turn, represses the activator. Turing demonstrated that when tuned appropriately, the nonlinear reaction kinetics and difference in diffusion coefficients can result in the formation of a stable periodic pattern in a field of initially homogenous signal, in which peaks of activator alternate with the inhibitor (*15*). This self-organizing system defines the spatial distribution of placodes and therefore the patterning of epithelial appendages. It is worth noting that in addition to RD, other factors such as mechanosensation of the tissue may be important for controlling skin appendage patterning (*16*). In this case, the patterning may still be via Turing instability, but using mechanical in addition to molecular RD interactions (*17*). We refer to this as a Turing-like system.

There is a growing body of experimental research supporting RD modeling throughout epithelial appendage development. This includes the role of RD in both patterning and morphogenesis of feathers and hair (*18*–*21*). These studies have revealed that molecular signals such as fibroblast growth factors (FGFs) and sonic hedgehog (*Shh*) can play autocatalytic activatory roles, whereas bone morphogenetic proteins (BMPs) can act as inhibitors (*18*, *22*). Despite evidence for RD patterning in classic tetrapod model organisms (i.e., mouse and chick), our understanding of this system in earlier diverging lineages is limited.

Chondrichthyans (cartilaginous fishes) occupy the sister lineage to osteichthyans (bony vertebrates) and constitute an earlier diverging lineage with respect to tetrapods. The elasmobranchs (sharks, skates, and rays) are a subclass of Chondrichthyes, which have hard, mineralized epithelial appendages known as odontodes. Odontodes include teeth and dermal denticles, which consist of a pulp cavity encased within layers of dentine and enameloid (*23*). It is thought that odontogenic competence originated in the dermal skeleton, giving rise to denticles as a precursor to the oral dentition of vertebrates (*24*–*26*). These structures have been observed in early vertebrates that lived as long as 450 million years ago (*27*, *28*). Denticles have evolved to fulfill a variety of functions, including provision of drag reduction and protective armor (*5*, *29*). It has previously been suggested that shark denticles do not follow a strict spatial pattern (*30*, *31*), although they do exhibit both intraspecific and interspecific variation in morphology and patterning, which closely relates to their function (*32*, *33*). Recent research has suggested that an RD mechanism may underlie the arrangement of denticles in a fossil adult Cretaceous shark (*Tribodus limae*) (*34*). However, experimental evidence addressing the initiation of patterning, and its genetic basis, is required to ascertain the role of this system in elasmobranchs.

Reif’s inhibitory field concept is considered the leading hypothesis for explaining odontode patterning (*35*). This theory describes how diffusion from existing odontodes can dictate the proximity of contemporaneous units, preventing placode formation within the perimeter of inhibition zones surrounding existing teeth or denticles (*35*, *36*). However, no underlying molecular basis has been identified to support this idea. In fact, it has been described as a verbal description of a restricted parameterization of an RD system (*34*).

**Fig. 1:**
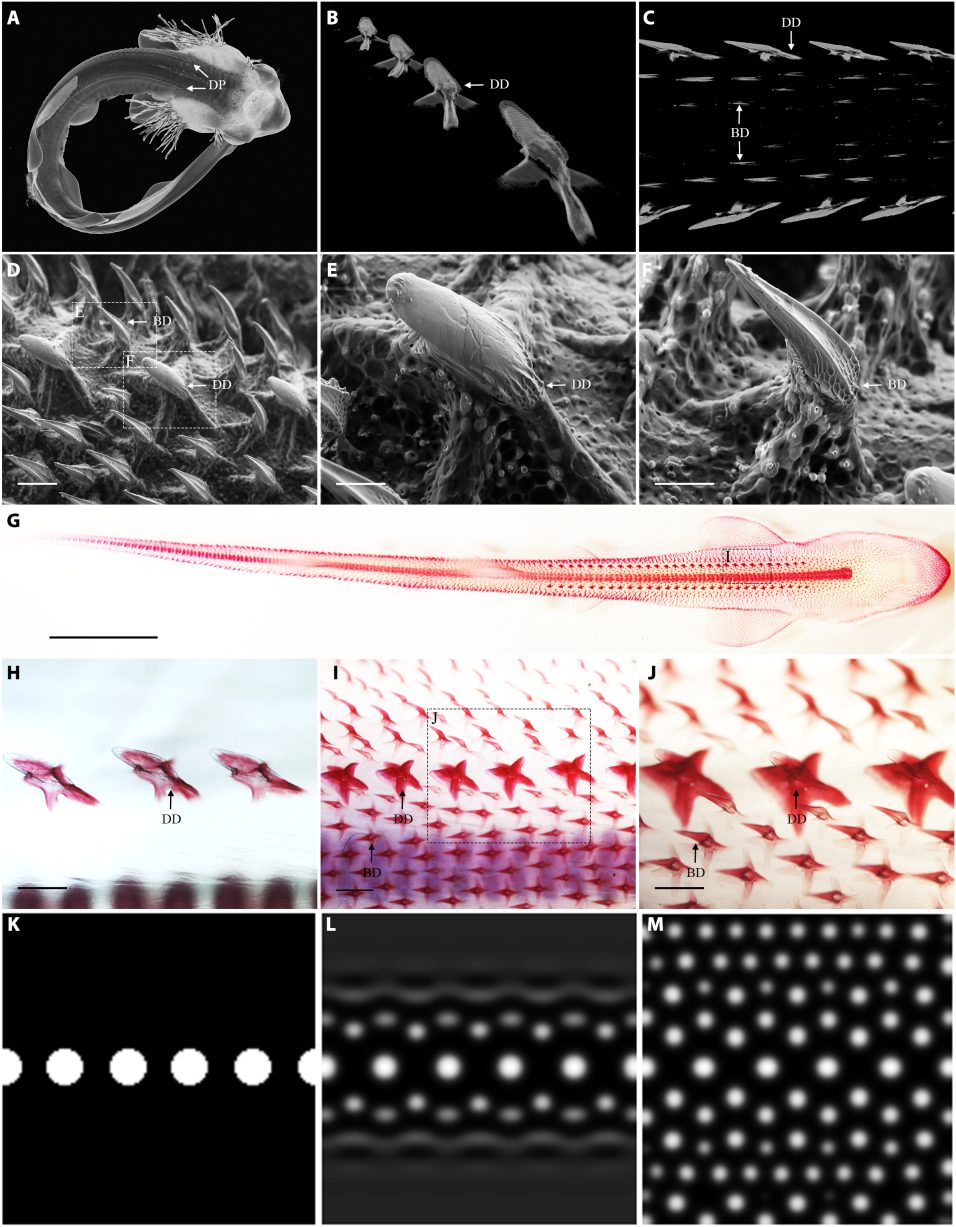
RD modeling can explain catshark denticle patterning. (**A**) Catsharks display two rows of dorsal denticle placodes (DP) at developmental stage 32 (~80 dpf). (**B** to **E** and **G** to **J**) These placodes undergo morphogenesis and mineralize to become dorsal denticles (DD). (C, D, **F**, and G to J) Their emergence precedes subsequent eruption of parallel, adjacent rows of body denticles (BD). Dorsal denticles also begin to mineralize (H) before body denticle development (I). Dorsal denticles are longer and broader than body denticles (E, F, and J). RD modeling suggests that diffusion and interaction of an activator and inhibitor from an initiator row representing dorsal denticles (**K**) can explain the patterning of surrounding body denticles (**L** and **M**). (A) to (C) are computed tomography (CT) scans, (D) to (F) are scanning electron microscopy (SEM) images, and (G) to (J) show alizarin red–stained samples. See Materials and Methods for details of RD modeling. Scale bars, 250 μm (D), 200 μm (E), 100 μm (F), 10 mm (G), and 400 μm (H to J).

There is thought to be early morphogenetic similarity between shark denticle and chick feather patterning, the latter of which is controlled by RD (*18*, *37*). Chick feathers initially develop sequentially in a dorsal longitudinal row along the embryo’s midline. This initiator row triggers subsequent placode formation in adjacent parallel rows until the integument is covered (*38*–*40*). This is consistent with an RD system (*8*, *18*). Embryonic sharks develop two dorsolateral rows of enlarged denticles that emerge before the subsequent eruption of intricately patterned body denticles (Fig. 1) (*36*, *41*, *42*). Soon after hatching, these rows are subsumed into general scalation (*26*). As observed during feather patterning (*18*, *39*), shark dorsal denticles may act as initiator rows that trigger the emergence of surrounding body denticles, following a conserved Turing-like system.

This study investigates epithelial appendage patterning in an early diverging lineage, with respect to tetrapods, using the small-spotted catshark (*Scyliorhinus canicula*). Using a combination of RD modeling and gene expression analysis, we investigate the mechanism and underlying molecular basis of shark denticle patterning. We then use small-molecule gene pathway inhibition experiments to reveal functional conservation of these genes. Last, we use RD modeling to demonstrate that our experimental results conform to a conserved Turing-like patterning system. Rather than following a random distribution (*30*), we find that shark denticle development is underpinned by a precise patterning mechanism that begins early in development. This conserved system may underlie the development of a broad range of epithelial appendages, thereby facilitating the evolution of diverse functional traits observed throughout vertebrates.

## RESULTS

### RD simulation and gene expression analyses suggest that a Turing-like system underlies shark body denticle patterning

We first investigated the morphogenetic patterning of shark denticles. Two rows of dorsal denticle placodes are visible at stage 32 of development [~80 days postfertilization (dpf)] (Fig. 1A) (*42*), preceding the emergence of body denticles (Fig. 1, C, D, and F). Compared to body denticles, dorsal denticles are larger and broader and do not have distinct ridges associated with hydrodynamic drag reduction (Fig. 1, D to F) (*5*). Simulation of an RD model was used to determine whether dorsal denticle rows can act as “initiator” rows, triggering the patterning of surrounding body denticles. Patterns were generated from a row of initiator spots representing dorsal denticles (Fig. 1K), from which waves of activatory and inhibitory morphogens radiated according to predefined values (Fig. 1L and table S1; see Materials and Methods for further details). Spots formed in rows adjacent and parallel to the initiator row. Upon reaching a steady state, initiator spots remained larger than newly formed spots (Fig. 1M), reflecting squamation of the shark (Fig. 1, D to J). This model provides theoretical support for a Turing-like system controlling denticle patterning in sharks.

**Fig. 2:**
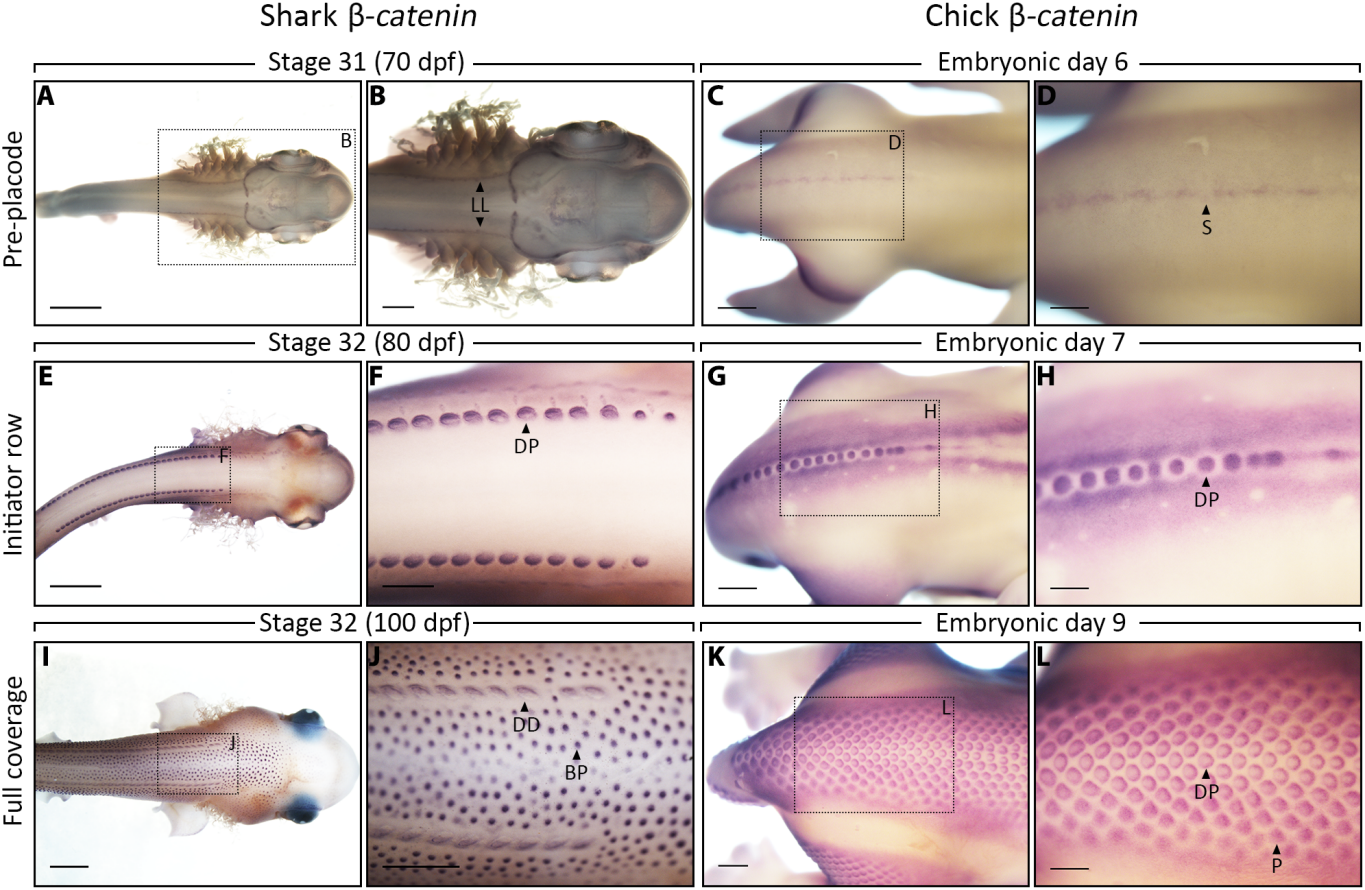
Conserved initiator rows may trigger surrounding epithelial placodes in the shark and chick. Whole-mount ISH for β-*cat* was undertaken throughout epithelial appendage patterning of shark denticles (**A**, **B**, **E**, **F**, **I**, and **J**) and chick feathers (**C**, **D**, **G**, **H**, **K**, and **L**). At E6, the chick displays a continuous stripe of β-*cat* expression (C and D), which then becomes compartmentalized into feather placodes (G and H). This initiator row triggers the emergence of surrounding feather placodes, following an RD system (*17*). (A and B) At stage 31 (~70 dpf), shark denticle placodes are not visible, although patterning of the lateral line sensory system is demarked by β-*cat*. (E and F) By stage 32 (~80 dpf), two dorsolateral rows of denticle placodes are visible. (I and J) Later in stage 32 (~100 dpf), surrounding rows of body denticle placodes also express β-*cat*. The shark dorsal denticle rows may be triggering body denticle emergence following a Turing-like system comparable to feather patterning. LL, lateral line; BP, body placode; P, placode. Scale bars, 2000 μm (A, E, and I), 1000 μm (B, C, G, J, and K), 500 μm (D, F, and H), and 750 μm (L).

To compare the patterning of shark denticles and chick feathers, we examined the expression of β-catenin (β-*cat*), an early regulator of chick epithelial placode signaling (Fig. 2 and fig. S1) (*43*). The chicken embryo expresses a dorsolateral stripe of β-*cat* at embryonic day 6 (E6) (Fig. 2, C and D). This stripe becomes compartmentalized into individual feather placodes at E7 (Fig. 2, G and H), which trigger the emergence of adjacent, parallel placode rows (Fig. 2, K and L) (*18*). The shark lateral line expresses β-*cat* at stage 31 (~70 dpf), shortly before denticle patterning begins (Fig. 2, A and B). A continuous stripe of expression was not observed in the shark; however, two dorsolateral rows of denticle placodes appeared simultaneously at stage 32 (~80 dpf), expressing β-*cat* (Fig. 2, E and F). These rows emerged parallel to either lateral line (Fig. 2, A to F). The smaller body denticle placodes subsequently emerged in rows adjacent to dorsal denticles later in stage 32 (~100 dpf) (Fig. 2, I and J). Shark dorsal denticles may be acting as initiator rows, triggering the emergence of surrounding units in a Turing-like mechanism comparable to feather patterning. Having noted this similarity between shark and chick epithelial appendage patterning, we next examined the expression of genes underlying a putative Turing-like patterning system in the shark.

**Fig. 3:**
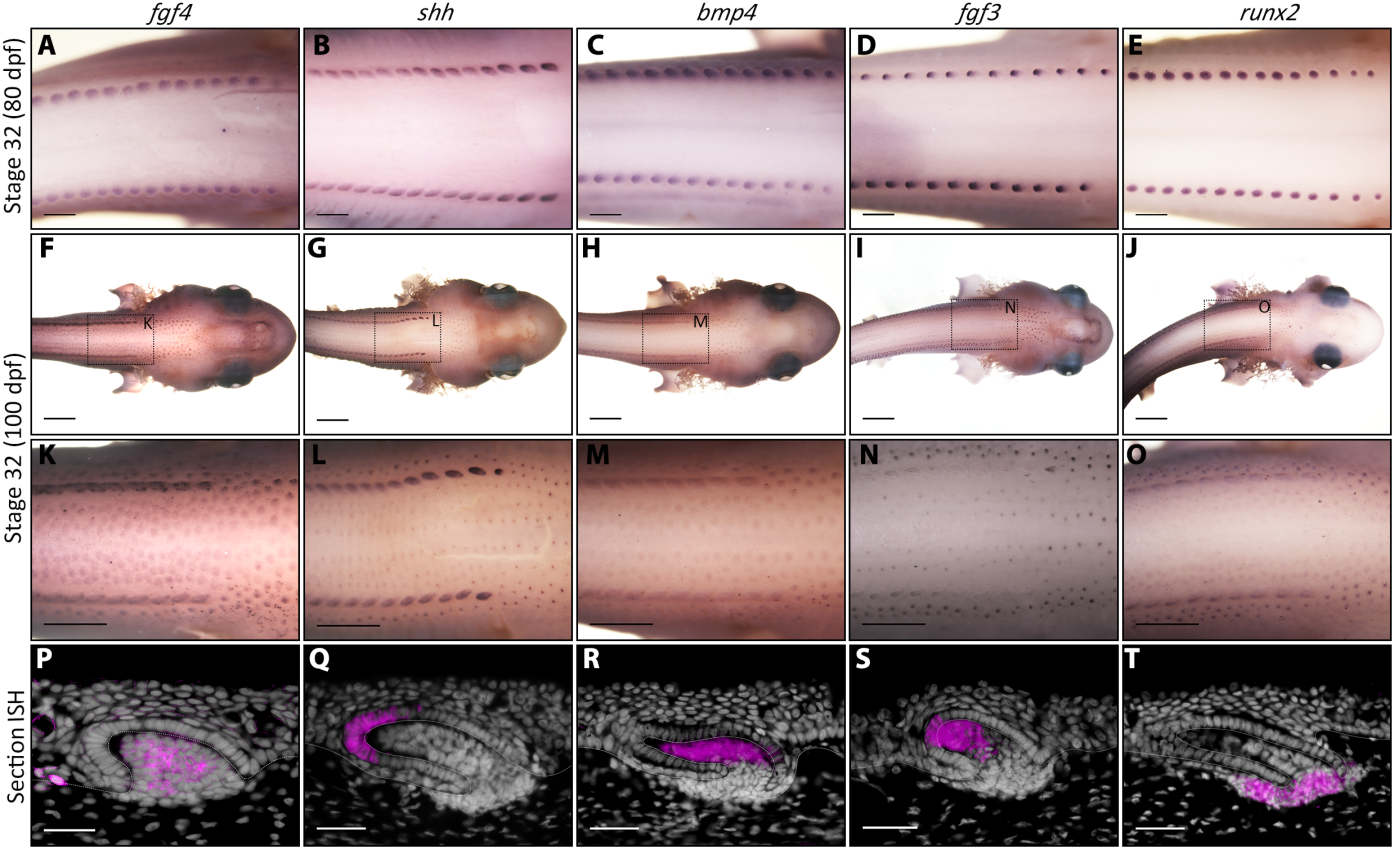
Conserved markers of RD are expressed during shark denticle patterning. The expression of genes thought to control RD patterning of chick feathers was charted during shark denticle patterning (*17*). (**A** to **C**) At stage 32 (~80 dpf), shark dorsal denticle placodes express *fgf4* and *shh*, which are considered activators of feather patterning, and *bmp4*, which is considered an inhibitor (*17*). (**D** and **E**) Dorsal rows also express *fgf3*, a dermal marker of feather bud development, and *runx2*, which is associated with FGF signaling during mammalian tooth development (*44*, *45*). (**F** to **O**) Later in stage 32 (~100 dpf), these genes are expressed during patterning of adjacent, parallel rows of body denticle placodes. (**P** to **R** and **T**) Section ISH of body denticles revealed epithelial expression of *shh* and mesenchymal expression of *fgf4*, *bmp4*, and *runx2*. (**S**) Expression of *fgf3* was observed in the epithelium and mesenchyme. White dashed lines separate columnar cells of the basal epithelium and the underlying mesenchyme. Scale bars, 500 μm (A to E), 2000 μm (F to J), 1000 μm (K to O), and 50 μm (P to T).

Using in situ hybridization (ISH), we sought to identify the potential activators and inhibitors comprising this Turing-like patterning system. A suite of genes were selected on the basis of their importance during feather patterning (*18*), and their expression was analyzed throughout squamation of the shark (Fig. 3 and fig. S1). At stage 31 (~70 dpf), dorsal denticle placodes were not detected (fig. S2), although β-*cat* expression labeled development of the lateral line sensory system (Fig. 2, A and B). By early stage 32 (~80 dpf), two dorsolateral rows of denticle placodes were visible, expressing the known activators of feather patterning, *fgf4* and *shh*, as well as the inhibitor *bmp4* (Fig. 3, A to C) (*18*, *42*). Similar to feather patterning, *bmp4* was expressed within placodes rather than the interplacode regions, suggesting that its inhibitory action is indirect (*18*). The mesenchymal marker of feather bud development, *fgf3*, was also expressed in dorsal denticle rows (Fig. 3D) (*44*), along with the runt domain transcription factor *runx2* (Fig. 3E), which is associated with FGF signaling throughout mammalian tooth morphogenesis and mineralization of other vertebrate skeletal elements (*45*–*47*). An anterior to posterior gradient of dorsal denticle development was noted.

Later in developmental stage 32 (~100 dpf), body denticle placodes become visible in rows adjacent and parallel to dorsal denticle rows. Body denticles extend throughout the ventral trunk and eventually propagate to the entire flank and ventral surface. We understand that there are multiple initiation sites (*48*), which are important for the extension of denticle patterning to the extremities, such as the paired pectoral fins. Redeployment of the same suite of genes expressed throughout dorsal denticle development was observed during patterning of these smaller body denticles (Fig. 3, F to O). Section ISH revealed that *shh* was expressed in the body denticle epithelium, whereas *fgf4*, *bmp4*, and *runx2* were expressed in the underlying mesenchyme (Fig. 3, P to R and T). The expression of *fgf3* was noted in both the epithelium and mesenchyme (Fig. 3S). Overall, these results revealed extensive conservation of RD-related gene expression between denticle and feather patterning (*18*, *43*, *49*).

### RD-related genes are functionally conserved during patterning of shark body denticles

**Fig. 4:**
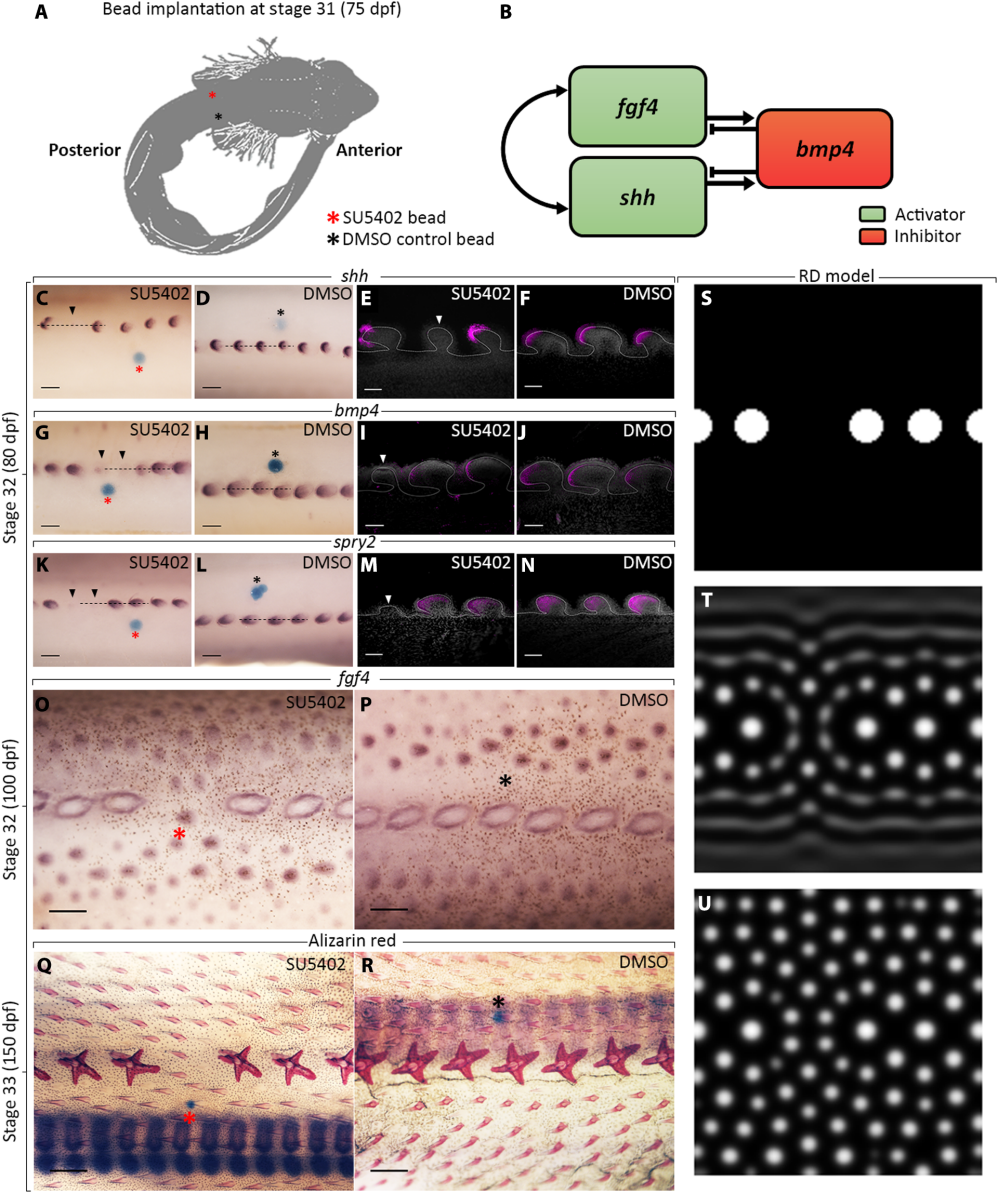
Bead inhibition experiments reveal functional conservation of RD-associated genes. (**A**) Beads loaded with the FGFR inhibitor SU5402 were implanted beneath the epithelium of shark embryos at 75 dpf. (**C** to **N**) First, we analyzed gene expression at 5 dpt. We propose that breaking a conserved activator-inhibitor feedback system between *fgf4*, *shh*, and *bmp4* (**B**) led to localized down-regulation of both *shh* and *bmp4*, resulting in stunted growth of dorsal denticle primordia, highlighted by black and white arrowheads (C to J) (*17*). (K to N) Expression of *spry2*, a transcriptional readout of FGF signaling, was also reduced (*50*). We observed localized inhibition of gene expression at 5 dpt in all SU5402 beaded samples (*n* = 5/5) and no DMSO control samples (*n* = 5/5). (**O**) Expression of *fgf4* at 25 dpt showed that this inhibition resulted in a gap in the dorsal denticle row, which became occupied by smaller body denticles (*n* = 2/2). (**P**) No gap was observed in DMSO control samples (*n* = 2/2). Alizarin red staining revealed that this gap was maintained in 75% of SU5402-treated dorsal rows at 50 dpt (*n* = 6/8), whereas no gap was observed in rows treated with DMSO control bead (*n* = 7/7) (fig. S5). (**Q**) This pattern was maintained in SU5402 beaded dorsal rows at 75 dpt, once body denticles had begun to mineralize (*n* = 7/8). (**R**) DMSO control samples did not show a gap (*n* = 9/9). The output of RD simulation including a gap in the initiator row (**S**) was consistent with the experimental patterning observed; smaller units occupied the gap in the row (**T** and **U**). Dashed black lines show the location of vibratome sections from whole-mount ISH (E, F, I, J, M, and N). Scale bars, 200 μm (C, D, G, H, K, and L), 50 μm (E, F, I, J, M, and N), 300 μm (O and P), and 400 μm (Q and R).

To verify the functional conservation of genes expressed during denticle patterning, we undertook small-molecule gene pathway inhibition experiments. Embryos were treated with beads loaded with either the FGF receptor inhibitor SU5402 (*50*) or dimethyl sulfoxide (DMSO) as a control. Beads were implanted beneath the epithelium in stage 31 embryos (~75 dpf), adjacent to rows of emerging dorsal denticle primordia (Fig. 4A). Development then continued before the genetic and phenotypic effects of treatment were examined at various time points.

First, ISH for RD-related genes was undertaken 5 days posttreatment (dpt). Localized inhibition of *shh* and *bmp4* expression was observed in dorsal denticle placodes treated with SU5402 beads, whereas the expression was unaltered in rows treated with DMSO beads (Fig. 4, C to J, and figs. S3 and S4, A to D). We propose that inhibition of FGF signaling disrupted a conserved activator-inhibitor feedback system between *fgf4, shh*, and *bmp4*, which similarly mediates feather patterning (Fig. 4B) (*18*). Furthermore, we observed down-regulation of sprouty 2 (*spry2*) expression (Fig. 4, K to N). As *spry2* is a downstream transcriptional readout of FGF signaling (*51*), this supports the idea that SU5402 treatment led to FGF inhibition in this system. Sections of whole-mount ISH samples revealed stunted development of denticle primordia (Fig. 4, C to N, and fig. S4), suggesting that inhibition of FGF signaling during early morphogenesis is sufficient to restrict dorsal denticle growth. As dorsal denticles develop in an anterior to posterior gradient, the treatment effect was strongest in the units undergoing early morphogenesis at the time of beading, rather than simply the units closest to the bead (Fig. 4, C to N). For example, in Fig. 4K, the bead is positioned anterior to units with reduced gene expression, as the units closest to the bead are more advanced in their development. Posterior units undergoing early morphogenesis (demarked with a black arrowhead) were affected by the treatment. Growth of the embryo may also affect proximity of the bead to the area of inhibition. These results suggest that there is functional conservation of a core gene regulatory network controlling shark denticle patterning, with FGF signaling playing an important activatory role.

Next, we examined the effect of the bead implants at 25 dpt, the stage at which smaller body denticles initiate (~100 dpf). Using ISH, we visualized *fgf4* expression to examine how the disruption of dorsal denticle development altered subsequent patterning (Fig. 4, O and P, and fig. S4, E and F). Dorsal denticle primordia failed to undergo morphogenesis following FGF inhibition, resulting in a gap in the row. This gap became infilled by smaller body denticle placodes (Fig. 4O), potentially as an inhibitory field surrounding dorsal denticles did not extend to this area. In contrast, control samples displayed a complete row of dorsal denticles (Fig. 4P). Alizarin red staining of SU5402 beaded samples fixed at 50 and 75 dpt revealed that this pattern was maintained throughout development, with smaller, mineralized body denticles occupying the gaps in the dorsal denticle rows (Fig. 4, Q and R, and figs. S5 and S6). Next, we examined whether this patterning response was consistent with an RD system. Therefore, we simulated the RD model (Fig. 1, K to M) with a unit missing from the initiator row (Fig. 4S) to mimic the functional experiment. The model output bore notable similarity to the pattern following bead implantation, with smaller units occupying the gap resulting from the missing initiator spot (Fig. 4, T and U). These results provide further evidence that a Turing-like system controls shark denticle patterning, as the model response remains robust following experimental manipulation.

### Retuning the RD model can explain the diversity of denticle patterning

**Fig. 5:**
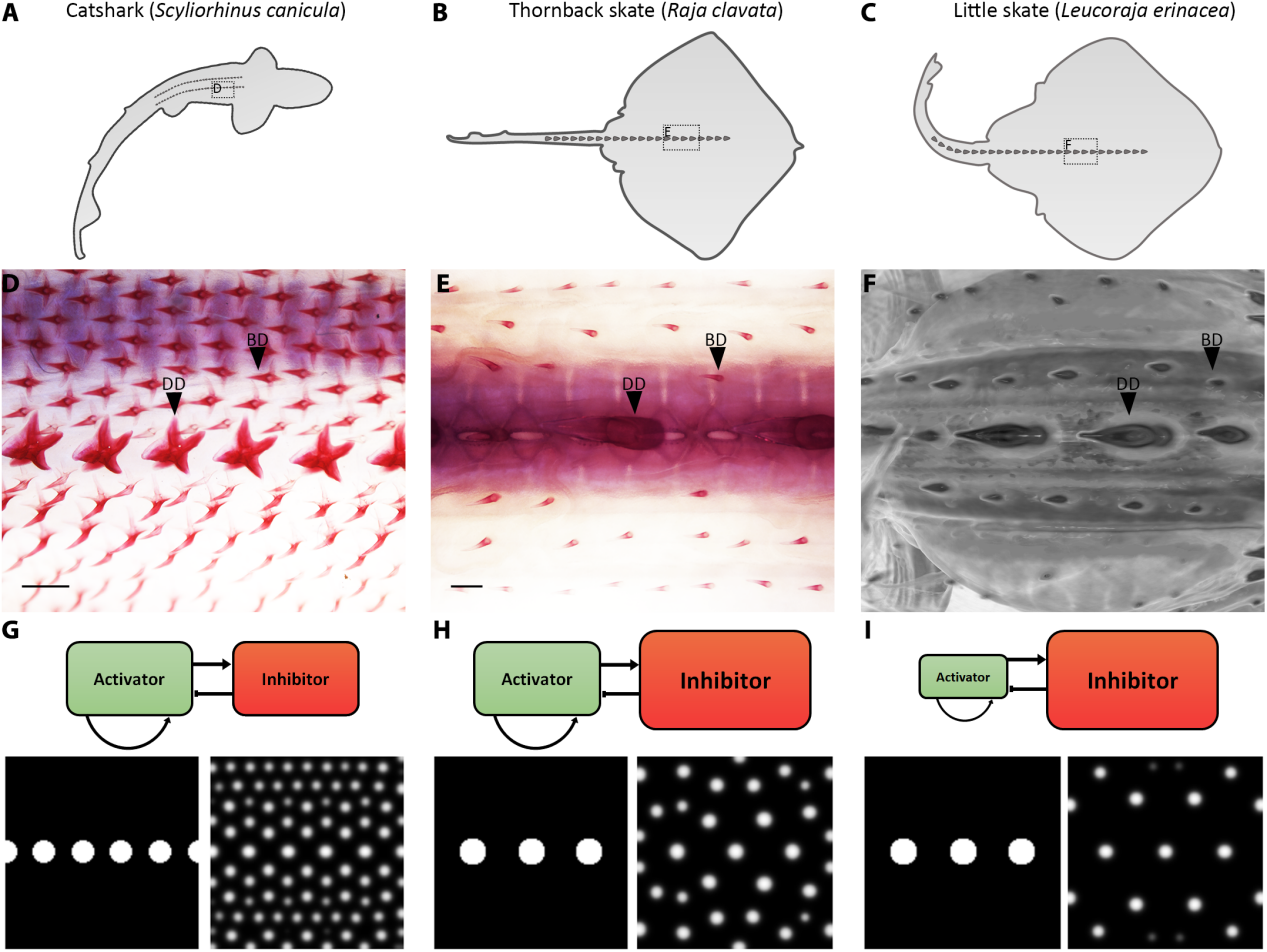
Alterations to RD parameter values can explain denticle patterning diversity. (**A** to **F**) Denticle diversity varies between elasmobranchs, with patterning becoming decreasingly dense from the catshark (*S. canicula*) to the thornback skate (*R. clavata*) and the little skate (*L. erinacea*). (**G**) Parameters of the RD model were initially set to result in catshark-like patterning. (**H**) Decreasing the inhibitor’s constitutive degradation rate (***d*_*v*_**) and maximum net production rate (***G*_max_**) while increasing its diffusion coefficient (***D*_*v*_**) resulted in a less dense thornback skate–like pattern. (E) Initiator spots were made larger and placed further apart to reflect the skate’s dorsal row. (**I**) Decreasing the activator’s constitutive production rate (***c*_*u*_**) further reduced coverage density, resulting in a little skate–like pattern. See Materials and Methods for details of RD modeling and table S1 for specific parameter values. Scale bars, 400 μm (D) and 1000 μm (E).

Having found evidence for Turing-like denticle patterning in the catshark, we sought to examine the role of this system in other elasmobranch species. Among elasmobranchs, denticle density is diverse, with most sharks having a relatively dense coverage. Comparatively, denticle coverage of the thornback skate (*Raja clavata*) and the little skate (*Leucoraja erinacea*) is increasingly sparse (Fig. 5, A to F). We retuned parameters of activatory and inhibitory morphogens in the RD model to predict this diversity in elasmobranch denticle density.

Model parameters were initially set to result in a catshark-like denticle pattern (Figs. 1, K to M, and 5, D and G). The inhibitor’s constitutive degradation rate (*d*_*v*_) and maximum net production rate (*G*_max_) were then decreased, while its diffusion coefficient (*D*_*v*_) was increased (table S1). Initiator spots were enlarged and spaced further apart to reflect the dorsal row of the skate (Fig. 5E). This led to decreased density of coverage, giving rise to patterning comparable to the thornback skate (Fig. 5, E and H). Next, the activator’s constitutive production rate (*c*_*u*_) was decreased (table S1). This further reduced the density of coverage, giving rise to patterning comparable to the little skate (Fig. 5, F to I). It is worth noting that numerous alternative combinations of parameter values could result in similar outputs to those shown here (Fig. 5, G to I), as well as outputs vastly more diverse (*9*). Overall, these results demonstrate that simple alterations to parameters of the RD model can give rise to a wide diversity of patterning outcomes comparable to those seen in extant elasmobranch species. The plasticity of this system may underlie broad variations covering the vast spectrum of vertebrate epithelial appendage patterns.

## DISCUSSION

Our results provide both theoretical and experimental evidence to suggest that shark denticle patterning is controlled by a conserved Turing-like system also known to mediate the feather patterning of chicks (*18*). This mechanism has likely controlled epithelial appendage development for at least 450 million years, spanning the evolution of vertebrates, from sharks to mammals (*9*, *21*, *28*). This system includes a dorsolateral initiator row that triggers the emergence of surrounding appendages, controlled by functionally conserved activators and inhibitors, including *fgf4*, *shh*, and *bmp4* (*18*). In addition, we show that altering the parameters of this system can explain denticle pattern diversity observed between different elasmobranch species.

Previous experimental work investigating RD patterning has broadly focused on its role throughout amniotes, specifically mice and chicks (*18*, *21*). In addition, the rearrangement of zebrafish pigmentation following partial stripe ablation is concurrent with an RD system (*52*). Denticle patterning following bead implantation bore notable similarity to this experiment (Fig. 4); in both systems, the gap in the original row was occupied by infilling from adjacent rows. We provide evidence for Turing-like patterning in chondrichthyans. This supports both experimental and theoretical work, suggesting that Turing patterning is of widespread importance throughout vertebrate evolutionary history and is common to taxonomically diverse vertebrate groups (*9*).

Furthermore, we demonstrate that alterations to the parameters of this system can explain the diversity of epithelial appendage patterns between different species (Fig. 5). Within elasmobranchs, this may have facilitated the evolution of various species-specific denticle functions, including protective armor, hydrodynamic drag reduction, feeding, and communication (*5*, *7*, *29*, *33*, *53*). More broadly, this system may underlie epithelial appendage patterns throughout other vertebrates. For example, RD may control mammalian hair density, which is closely linked to thermoregulation (*6*). Small changes to this conserved system may underpin pattern diversity throughout vertebrates.

Future research should address the formation of the initiator rows that trigger subsequent Turing patterning (Fig. 2). In the chick, this row originates as a continuous stripe, which then bifurcates into two rows, before the expression becomes localized to individual feather placodes (*54*). The shark has two initiator rows of denticle placodes (*18*, *39*), suggesting the single bifurcating initiator row of the chick may be a derived feature. Transcriptome sequencing has shown that genes associated with neural development are significantly up-regulated in the skin during patterning of the chick initiator row. This is indicative of developmental synchronicity between the nervous system and feather patterning (*55*). The shark lateral line is a system of innervated sensory organs that appear parallel to subsequent dorsal row placodes (Fig. 2, A and B, and fig. S2D). It is possible that these systems are synchronous in the shark, with the lateral line mediating the patterning of the shark denticle initiator row. Furthermore, the lateral line extends the entire length of the body and may mediate Turing-like patterning posterior to the dorsal rows, which extend approximately half way along the dorsal trunk. In addition, there are multiple sites of pattern initiation, including those located on the wings and pectoral fins of the chick and elasmobranchs, respectively (*48*, *56*). Whether these sites have individual initiator rows is unknown, presenting a gap in our understanding of pattern initiation.

The importance of RD-controlled patterning has long been debated (*9*). However, there is a growing body of both theoretical and experimental work supporting the relevance of this model (*11*, *12*, *18*–*21*). Our findings provide support for this research, demonstrating that an ancient Turing-like system controls epithelial appendage patterning in chondrichthyans, which belong to an early diverging lineage, with respect to tetrapods. We suggest that diverse vertebrate groups share this common, conserved patterning mechanism, before deviation in later morphogenesis gives rise to clade-specific integumentary appendages, such as denticles, feathers, and hair.

## MATERIALS AND METHODS

### Shark and chick husbandry

The University of Sheffield is a licensed establishment under the Animals (Scientific Procedures) Act 1986. All animals were culled by approved methods cited under Schedule 1 to the Act. Fertilized Bovan brown chicken eggs (Henry Stewart & Co., Norfolk, UK) were incubated at 37.5°C before overnight fixation in Carnoy’s solution between E6 and E9. *S. canicula* embryos (North Wales Biologicals, Bangor, UK) were raised in oxygenated artificial saltwater (Instant Ocean) at 16°C. Shark embryos were culled with MS-222 (tricaine) at 300 mg/liter and fixed overnight in 4% paraformaldehyde in phosphate-buffered saline (PBS). After fixation, chicken and shark embryos were dehydrated through a graded series of PBS to ethanol (EtOH) and stored at −20°C.

### Micro-CT and SEM

High-resolution micro-CT scanning was conducted using an Xradia Micro-XCT scanner at the Imaging and Analysis Centre, Natural History Museum, London. *S. canicula* embryos were stained with 0.1% phosphotungstic acid in 70% EtOH for 3 days to enhance contrast. Scans were rendered using the three-dimensional volume exploration tool Drishti (https://github.com/nci/drishti). SEM was undertaken using a Hitachi TM3030Plus Benchtop SEM scanner at 15,000 V.

### Alizarin red clear and staining

Embryos were rehydrated from EtOH to PBS and stained overnight with alizarin red in potassium hydroxide (KOH), as previously described (*4*). Samples were imaged in glycerol using a Nikon SMZ15000 stereomicroscope. Scale bars were created in Fiji (*57*).

### RD modeling

RD modeling of shark body denticle patterning was undertaken using an activator-inhibitor model proposed by Kondo and Miura (*9*) based on the equations

$$\frac{\partial u}{\partial t}=F(u,v)-d_{u}u+D_{u}\Delta u$$ (1)

$$\frac{\partial v}{\partial t}=G(u,v)-d_{v}v+D_{v}\Delta v$$ (2)

where *u*(*t*, *x*, *y*) and *v*(*t*, *x*, *y*) denote the concentrations of an activator and inhibitor, respectively, at time *t* and location (*x*, *y*). Equations 1 and 2 describe the rate of change of these concentrations in time and space due to diffusion and regulated production and degradation of the molecular species. The nonlinear functions *F*(*u*, *v*) and *G*(*u*, *v*) are defined by

$$F(u,v)=\{\begin{array}{cc} 0 & \text{if}\;a_{u}u+b_{u}v+c_{u}<0,\\ F_{\text{max}} & \text{if}\;a_{u}u+b_{u}v+c_{u}>F_{\text{max}},\\ a_{u}u+b_{u}v+c_{u} & \text{otherwise}, \end{array}$$ (3)

$$G(u,v)=\{\begin{array}{cc} 0 & \text{if}\;a_{v}u+b_{v}v+c_{v}<0,\\ G_{\text{max}} & \text{if}\;a_{v}u+b_{v}v+c_{v}>G_{\text{max}},\\ a_{v}u+b_{v}v+c_{v} & \text{otherwise}. \end{array}$$ (4)

Equations 1 and 2 were solved in the two-dimensional square domain 0 < *x* < *L*, 0 < *y* < *L* for times 0 < *t* < *T* subject to no-flux boundary conditions and prescribed initial conditions that varied across simulations. For the simulations shown in Figs. 1 (K to M) and 5 (G to I), the initial condition was given by

$$u(0,x,y)=\{\begin{array}{cc} u_{0} & \text{if}\;{\left(x-x_{i}\right)}^{2}+{\left(y-y_{i}\right)}^{2}<{\left(R_{\text{spot}}\right)}^{2}\;\text{for}\;i\in \{0,\ldots ,n_{\text{spot}}-1\},\\ 0 & \text{otherwise}, \end{array}$$ (5)

v(0,x,y)=0 (6)

where each (*x*_*i*_, *y*_*i*_) defines the center of a spot in an initiator row representing dorsal denticles of a given number (*n*_spot_) and radius (*R*_spot_). Figures 1 (K to M) and 5G were generated using *R*_spot_ = 4.5, *n*_spot_ = 6, and (*x*_*i*_, *y*_*i*_) = (*iL*/5, *L*/2). Figure 4 (S to U) was generated using the same initial condition but with the spot centered at (*x*_2_, *y*_2_) removed. Figure 5H was generated using *R*_spot_ = 5.25, *n*_spot_ = 3, and (*x*_*i*_, *y*_*i*_) = ((3*i* + 2)*L*/10, *L*/2), reflecting fewer, larger, more widely spaced initiator spots.

The RD model was solved numerically using an explicit finite difference method, choosing a spatial discretization Δ*x* and sufficiently small time step Δ*t* to ensure numerical stability. Python code to generate Figs. 1 (K to M), 4 (S to U), and 5 (G to I) is provided in the Supplementary Materials. The parameter values used to generate Figs. 1 (K to M) and 4 (S to U) were given by *d*_*u*_ = 0.03, *D*_*u*_ = 0.02, *a*_*u*_ = 0.08, *b*_*u*_ = − 0.08, *c*_*u*_ = 0.04, *F*_max_ = 0.2, *d*_*v*_ = 0.08, *D*_*v*_ = 0.6, *a*_*v*_ = 0.16, *b*_*v*_ = 0, *c*_*v*_ = − 0.05, and *G*_max_ = 0.5, with a domain of size *L* = 75, end time *T* = 1500, spot radius *R* = 4.5, initial concentration *u*_0_ = 5, and discretization Δ*x* = *L*/128 ≈ 0.58, Δ*t* = (Δ*x*)^2^/8*D*_*v*_ ≈ 0.07. These values were chosen on the basis of an ad hoc exploration of parameter space around parameter values previously identified by Kondo and Miura as leading to patterning (*9*). Parameter values for Fig. 5 are given in table S1. For Fig. 5 (H and I), because the value of *D*_*v*_ was reduced, we updated the value of Δ*t* = (Δ*x*)^2^/8*D*_*v*_ ≈ 0.04 to maintain numerical stability.

### In situ hybridization

Digoxigenin-labeled antisense riboprobes were designed using partial skate (*L. erinacea*) and catshark (*S. canicula*) EST (expressed sequence tag) assemblies (SkateBase; skatebase.org) (*58*), the Vertebrate TimeCapsule (VTcap; transcriptome.cdb.riken.go.jp/vtcap), and transcriptome data from RNA sequencing (unpublished). Sequences of forward and reverse primers (Sigma) are as follows: chick β-*cat*, TCTCACATCACCGTGAAGGC (forward) and CCTGATGTCTGCTGGTGAGG (reverse); shark β-*cat*, GGTGAAAATGCTTGGGTCT (forward) and GGACAAGGGTTCCTAGAAGA (reverse); shark *fgf4*, ATGTTGATCAGGAAGCTGCG (forward) and GTATGCGTTGGATTCGTAGGC (reverse); shark *shh*, TGACTCCCAATTACAACCCGG (forward) and TCAGGTCCTTCACTGACTTGC (reverse); shark *bmp4*, GATCTCTACAGGCTGCAGTCC (forward) and GATCTCTACAGGCTGCAGTCC (reverse); shark *fgf3*, CTTGCTCAACAGTCTTAAGTTATGG (forward) and CGGAGGAGGCTCTACTGTG (reverse); shark *runx2*, ATCTCTCAATCCTGCACCAGC (forward) and CCAGACAGACTCATCAATCCTCC (reverse); and shark *spry2*, AACTAGCACTGTGAGTAGCGG (forward) and GTTCCGAGGAGGTAAACTGGG (reverse). Riboprobes were synthesized using the Riboprobe System SP6/T7 Kit (Promega) and DIG RNA Labeling Mix (Roche). Whole-mount and section ISH was performed as previously described (*4*, *59*). To compare sequences between the chick and shark, phylogenetic gene trees were reconstructed from protein coding sequences extracted from www.ensemble.org, aligned to *S. canicula* sequences obtained during probe synthesis (see fig. S1 for details) (*60*, *61*). Whole-mount ISH samples were imaged using a Nikon SMZ15000 stereomicroscope, and sections were imaged using an Olympus BX51 microscope and Olympus DP71 Universal digital camera attachment. Vibratome sections shown in Fig. 4 were cut at a thickness of 30 μm. Adjustments to image contrast and brightness were made to improve clarity. Scale bars were added using Fiji (*57*).

### Bead implantation experiments

Embryos were treated with Affi-Gel Blue beads (Bio-Rad) loaded with SU5402 (2 mg/ml; Sigma) in DMSO. Control beads were loaded with DMSO. Stage 31 (~75 dpf) embryos were removed from their egg cases and anaesthetized before beads were surgically implanted using sharpened tungsten wire. Embryos were then cultured in six-well plates with artificial salt water and 1% penicillin-streptomycin (Thermo Fisher Scientific). At stage 32 (~100 dpf), embryos were transferred to 70-ml plastic containers (Sarstedt) floating in a 200-liter tank. The number of replicates and observed effects for different analyses are shown in Table 1.

**Table 1:** Summary of the number of replicates for bead inhibition experiments (shown in Fig. 4 and figs. S4 and S5).

**Stage fixed** **(dpf)**	**Analysis** **type**	**SU5402 bead** **(number of** **dorsal rows** **affected/total)**	**DMSO control** **bead (number** **of dorsal rows** **unaffected/total)**
80	ISH	5/5	5/5
100	ISH	2/2	2/2
125	Alizarin red	6/8	7/7
150	Alizarin red	7/8	9/9
	Total	20/23 = 87%	23/23 = 100%

## Supplementary Material

http://advances.sciencemag.org/cgi/content/full/4/11/eaau5484/DC1

## SUPPLEMENTARY MATERIALS

Supplementary material for this article is available at http://advances.sciencemag.org/cgi/content/full/4/11/eaau5484/DC1 Fig. S1. Phylogenetic gene trees reconstructed from protein coding sequences extracted from www.ensembl.org. Fig. S2. Dorsal denticle placodes are not visible at stage 31 (~70 dpf). Fig. S3. Individual vibratome section images comprising false-colored ISH composite images. Fig. S4. Replicates of beaded shark embryos after whole-mount ISH. Fig. S5. Replicates of clear and stained shark embryos showing RD response to SU5402 beading. Fig. S6. SEM images of shark embryo 75 days after beading. Table S1. Activator and inhibitor values for RD model. Python script for RD simulations
