# Supplementary material for: An ancient Turing-like patterning mechanism regulates skin denticle development in sharks
Source: Sci Adv. 2018 Nov 7;4(11):eaau5484. doi: 10.1126/sciadv.aau5484 (PMC6221541; doi:10.1126/sciadv.aau5484)
Supplement: http://advances.sciencemag.org/cgi/content/full/4/11/eaau5484/DC1 [file supp_4_11_eaau5484__index.html]

Science Advances | Science Advances

## Supplementary Materials

**The PDF file includes:**

- Fig. S1. Phylogenetic gene trees reconstructed from protein coding sequences extracted from www.ensembl.org.
- Fig. S2. Dorsal denticle placodes are not visible at stage 31 (~70 dpf).
- Fig. S3. Individual vibratome section images comprising false-colored ISH composite images.
- Fig. S4. Replicates of beaded shark embryos after whole-mount ISH.
- Fig. S5. Replicates of clear and stained shark embryos showing RD response to SU5402 beading.
- Fig. S6. SEM images of shark embryo 75 days after beading.
- Table S1. Activator and inhibitor values for RD model.

Download PDF

**Other Supplementary Material for this manuscript includes the following:**

- Python script for RD simulations (.py format)

**Files in this Data Supplement:**

- Adobe PDF - aau5484\_SM.pdf
