# Supplementary material for: An ancient Turing-like patterning mechanism regulates skin denticle development in sharks
Source: Sci Adv. 2018 Nov 7;4(11):eaau5484. doi: 10.1126/sciadv.aau5484 (PMC6221541; doi:10.1126/sciadv.aau5484)
Supplement: http://advances.sciencemag.org/cgi/content/full/4/11/eaau5484/DC1 [file aau5484_SM.pdf]

Science Advances
AAAS

advances.sciencemag.org/cgi/content/full/4/11/eaau5484/DC1

Supplementary Materials for

An ancient Turing-like patterning mechanism regulates skin denticle development in sharks

Rory L. Cooper, Alexandre P. Thiery, Alexander G. Fletcher, Daniel J. Delbarre, Liam J. Rasch, Gareth J. Fraser*

*Corresponding author. Email: g.fraser@ufl.edu

Published 7 November 2018, *Sci. Adv.* **4**, eaau5484 (2018)
DOI: 10.1126/sciadv.aau5484

The PDF file includes:

Fig. S1. Phylogenetic gene trees reconstructed from protein coding sequences extracted from www.ensembl.org.
Fig. S2. Dorsal denticle placodes are not visible at stage 31 (~70 dpf).
Fig. S3. Individual vibratome section images comprising false-colored ISH composite images.
Fig. S4. Replicates of beaded shark embryos after whole-mount ISH.
Fig. S5. Replicates of clear and stained shark embryos showing RD response to SU5402 beading.
Fig. S6. SEM images of shark embryo 75 days after beading.
Table S1. Activator and inhibitor values for RD model.

Other Supplementary Material for this manuscript includes the following:

(available at advances.sciencemag.org/cgi/content/full/4/11/eaau5484/DC1)

Python script for RD simulations (.py format)

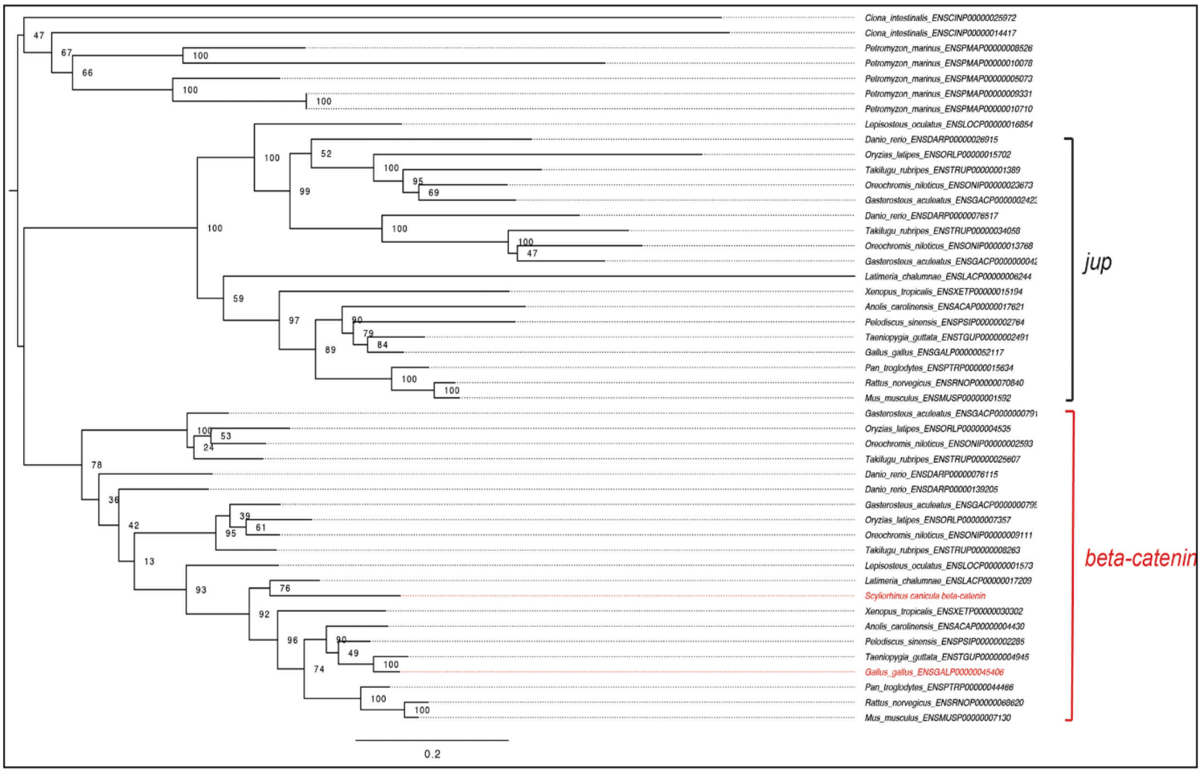
Ciona_intestinalis_ENSCINP00000025972
Ciona_intestinalis_ENSCINP00000014417
Petromyzon_marinus_ENSPMAP00000008526
Petromyzon_marinus_ENSPMAP00000010078
Petromyzon_marinus_ENSPMAP00000005073
Petromyzon_marinus_ENSPMAP00000009331
Petromyzon_marinus_ENSPMAP00000010710
Lepisosteus_oculatus_ENSLOCP00000016854
Danio_rerio_ENSDARP00000026915
Oryzias_latipes_ENSORLP00000015702
Takifugu_rubripes_ENSTRUP00000001389
Oreochromis_niloticus_ENSONIP00000023673
Gasterosteus_aculeatus_ENSGACP0000002423
Danio_rerio_ENSDARP00000076517
Takifugu_rubripes_ENSTRUP00000034058
Oreochromis_niloticus_ENSONIP00000013768
Gasterosteus_aculeatus_ENSGACP000000004
Latimeria_chalumnae_ENSLACP00000006244
Xenopus_tropicalis_ENSXETP00000015194
Anolis_carolinensis_ENSACAP00000017621
Pelodiscus_sinensis_ENSPSIP00000002764
Taeniopygia_guttata_ENSTGUP00000002491
Gallus_gallus_ENSGALP00000052117
Pan_troglodytes_ENSPTRP00000015634
Rattus_norvegicus_ENSRNOP00000070840
Mus_musculus_ENSMUSP00000001592
Gasterosteus_aculeatus_ENSGACP000000079
Oryzias_latipes_ENSORLP00000004535
Oreochromis_niloticus_ENSONIP00000002593
Takifugu_rubripes_ENSTRUP00000025607
Danio_rerio_ENSDARP00000076115
Danio_rerio_ENSDARP00000139205
Gasterosteus_aculeatus_ENSGACP000000079
Oryzias_latipes_ENSORLP00000007357
Oreochromis_niloticus_ENSONIP00000009111
Takifugu_rubripes_ENSTRUP00000008263
Lepisosteus_oculatus_ENSLOCP00000001573
Latimeria_chalumnae_ENSLACP00000017209
Scyliorhinus canicula beta-catenin
Xenopus_tropicalis_ENSXETP00000030302
Anolis_carolinensis_ENSACAP00000004430
Pelodiscus_sinensis_ENSPSIP00000002285
Taeniopygia_guttata_ENSTGUP00000004945
Gallus_gallus_ENSGALP00000045406
Pan_troglodytes_ENSPTRP00000044466
Rattus_norvegicus_ENSRNOP00000068620
Mus_musculus_ENSMUSP00000007130
jup
beta-catenin
0.2

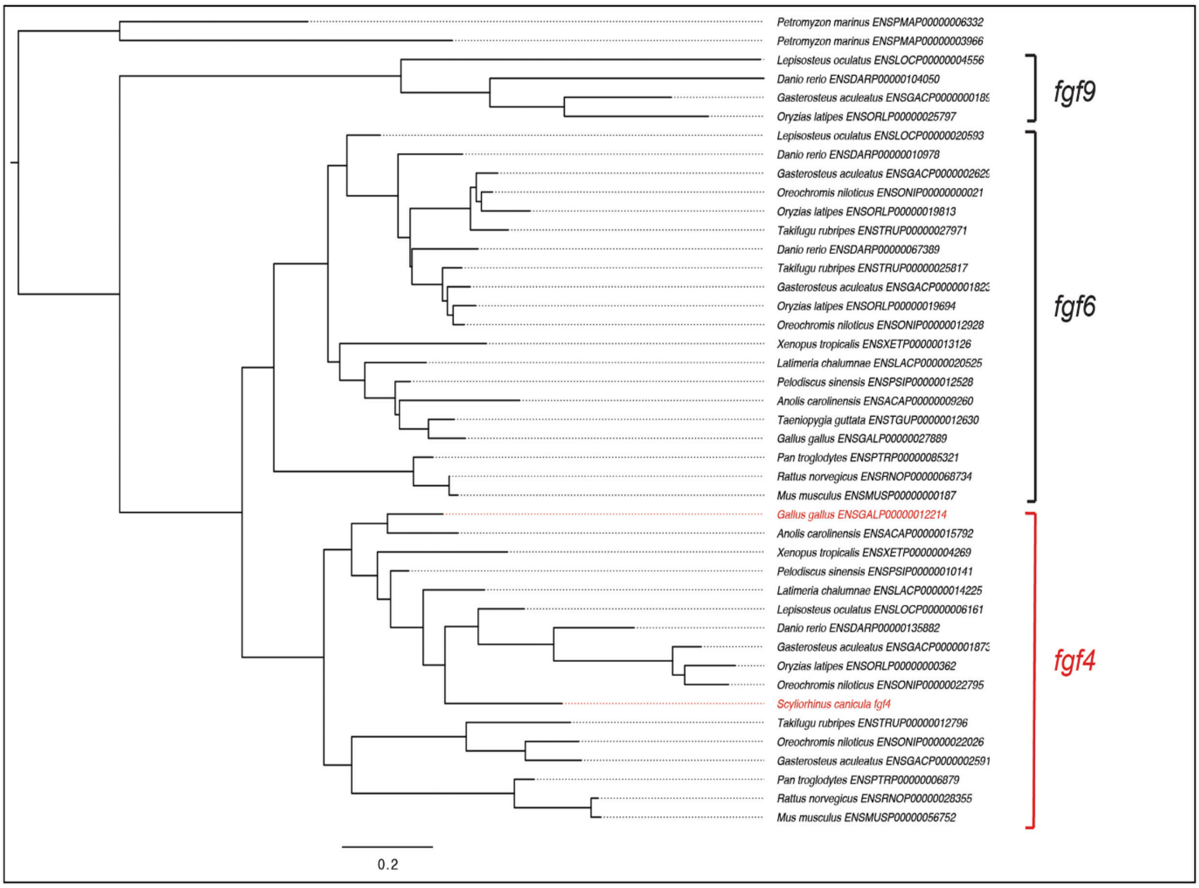
Petromyzon marinus ENSPMAP00000006332
Petromyzon marinus ENSPMAP00000003966
Lepisosteus oculatus ENSLOCP00000004556
Danio rerio ENSDARP00000104050
Gasterosteus aculeatus ENSGACP000000018
Oryzias latipes ENSORLP00000025797
Lepisosteus oculatus ENSLOCP00000020593
Danio rerio ENSDARP00000010978
Gasterosteus aculeatus ENSGACP000000262
Oreochromis niloticus ENSONIP00000000021
Oryzias latipes ENSORLP00000019813
Takifugu rubripes ENSTRUP00000027971
Danio rerio ENSDARP00000067389
Takifugu rubripes ENSTRUP00000025817
Gasterosteus aculeatus ENSGACP000000182
Oryzias latipes ENSORLP00000019694
Oreochromis niloticus ENSONIP00000012928
Xenopus tropicalis ENSXETP00000013126
Latimeria chalumnae ENSLACP00000020525
Pelodiscus sinensis ENSPSIP00000012528
Anolis carolinensis ENSACAP00000009260
Taeniopygia guttata ENSTGUP00000012630
Gallus gallus ENSGALP00000027889
Pan troglodytes ENSPTRP00000085321
Rattus norvegicus ENSRNOP00000068734
Mus musculus ENSMUSP00000000187
Gallus gallus ENSGALP00000012214
Anolis carolinensis ENSACAP00000015792
Xenopus tropicalis ENSXETP00000004269
Pelodiscus sinensis ENSPSIP00000010141
Latimeria chalumnae ENSLACP00000014225
Lepisosteus oculatus ENSLOCP00000006161
Danio rerio ENSDARP00000135882
Gasterosteus aculeatus ENSGACP000000187
Oryzias latipes ENSORLP00000000362
Oreochromis niloticus ENSONIP00000022795
Scyliorhinus canicula fgf4
Takifugu rubripes ENSTRUP00000012796
Oreochromis niloticus ENSONIP00000022026
Gasterosteus aculeatus ENSGACP000000259
Pan troglodytes ENSPTRP00000006879
Rattus norvegicus ENSRNOP00000028355
Mus musculus ENSMUSP00000056752
fgf9
fgf6
fgf4
0.2

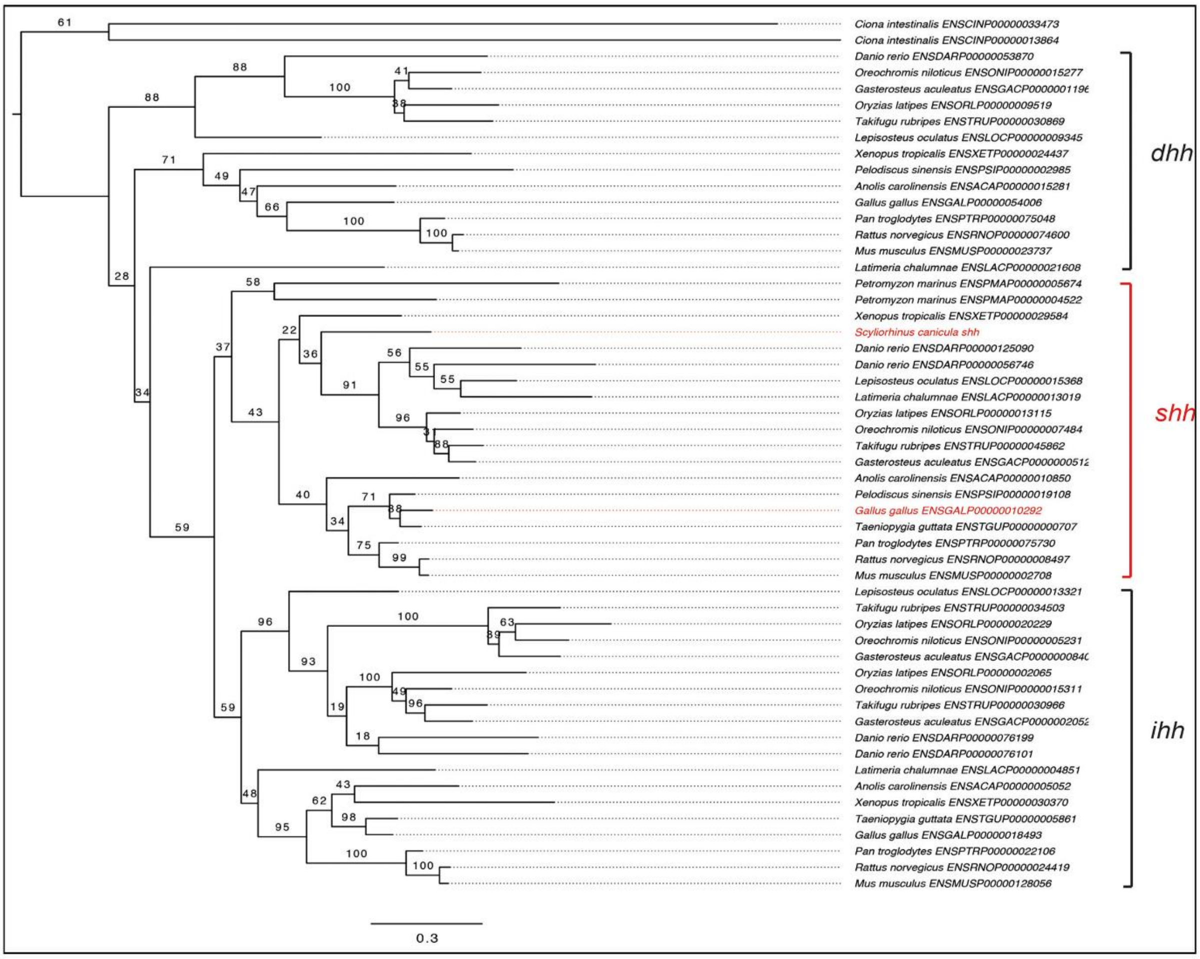

Ciona intestinalis ENSCINP00000033473
Ciona intestinalis ENSCINP00000013864
Danio rerio ENSDARP00000053870
Oreochromis niloticus ENSONIP00000015277
Gasterosteus aculeatus ENSGACP0000001196
Oryzias latipes ENSORLP00000009519
Takifugu rubripes ENSTRUP00000030869
Lepisosteus oculatus ENSLOCP00000009345
Xenopus tropicalis ENSXETP00000024437
Pelodiscus sinensis ENSPSIP00000002985
Anolis carolinensis ENSACAP00000015281
Gallus gallus ENSGALP00000054006
Pan troglodytes ENSPTRP00000075048
Rattus norvegicus ENSRNOP00000074600
Mus musculus ENSMUSP00000023737
Latimeria chalumnae ENSLACP00000021608
Petromyzon marinus ENSPMAP00000005674
Petromyzon marinus ENSPMAP00000004522
Xenopus tropicalis ENSXETP00000029584
Scyliorhinus canicula shh
Danio rerio ENSDARP00000125090
Danio rerio ENSDARP00000056746
Lepisosteus oculatus ENSLOCP00000015368
Latimeria chalumnae ENSLACP00000013019
Oryzias latipes ENSORLP00000013115
Oreochromis niloticus ENSONIP00000007484
Takifugu rubripes ENSTRUP00000045862
Gasterosteus aculeatus ENSGACP0000000512
Anolis carolinensis ENSACAP00000010850
Pelodiscus sinensis ENSPSIP00000019108
Gallus gallus ENSGALP00000010292
Taeniopygia guttata ENSTGUP00000000707
Pan troglodytes ENSPTRP00000075730
Rattus norvegicus ENSRNOP00000008497
Mus musculus ENSMUSP00000002708
Lepisosteus oculatus ENSLOCP00000013321
Takifugu rubripes ENSTRUP00000034503
Oryzias latipes ENSORLP00000020229
Oreochromis niloticus ENSONIP00000005231
Gasterosteus aculeatus ENSGACP0000000840
Oryzias latipes ENSORLP00000002065
Oreochromis niloticus ENSONIP00000015311
Takifugu rubripes ENSTRUP00000030966
Gasterosteus aculeatus ENSGACP0000002052
Danio rerio ENSDARP00000076199
Danio rerio ENSDARP00000076101
Latimeria chalumnae ENSLACP00000004851
Anolis carolinensis ENSACAP00000005052
Xenopus tropicalis ENSXETP00000030370
Taeniopygia guttata ENSTGUP00000005861
Gallus gallus ENSGALP00000018493
Pan troglodytes ENSPTRP00000022106
Rattus norvegicus ENSRNOP00000024419
Mus musculus ENSMUSP00000128056
dhh
shh
ihh
0.3

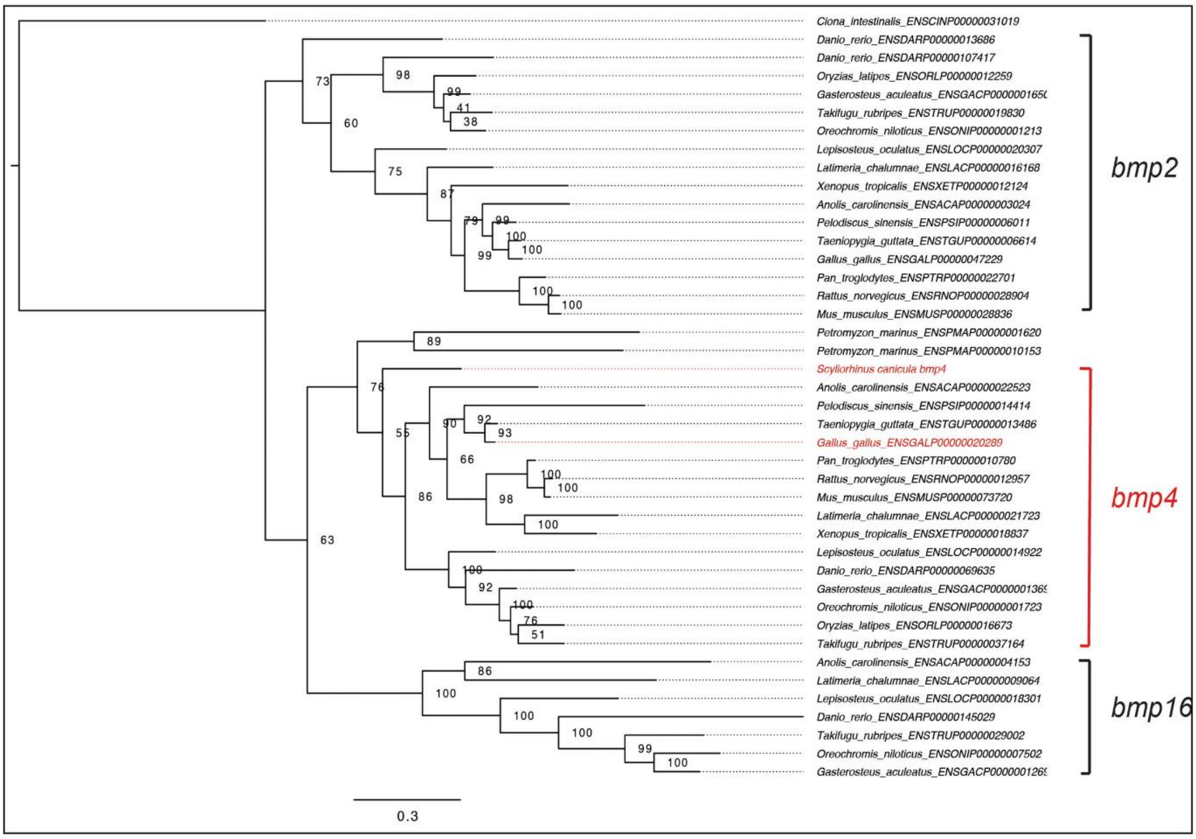

Ciona_intestinalis_ENSCINP00000031019
Danio_rerio_ENSDARP00000013686
Danio_rerio_ENSDARP00000107417
Oryzias_latipes_ENSORLP00000012259
Gasterosteus_aculeatus_ENSGACP0000001650
Takifugu_rubripes_ENSTRUP00000019830
Oreochromis_niloticus_ENSONIP00000001213
Lepisosteus_oculatus_ENSLOCP00000020307
Latimeria_chalumnae_ENSLACP00000016168
Xenopus_tropicalis_ENSXETP00000012124
Anolis_carolinensis_ENSACAP00000003024
Pelodiscus_sinensis_ENSPSIP00000006011
Taeniopygia_guttata_ENSTGUP00000006614
Gallus_gallus_ENSGALP00000047229
Pan_troglodytes_ENSPTRP00000022701
Rattus_norvegicus_ENSRNOP00000028904
Mus_musculus_ENSMUSP00000028836
Petromyzon_marinus_ENSPMAP00000001620
Petromyzon_marinus_ENSPMAP00000010153
Scyliorhinus canicula bmp4
Anolis_carolinensis_ENSACAP00000022523
Pelodiscus_sinensis_ENSPSIP00000014414
Taeniopygia_guttata_ENSTGUP00000013486
Gallus_gallus_ENSGALP00000020289
Pan_troglodytes_ENSPTRP00000010780
Rattus_norvegicus_ENSRNOP00000012957
Mus_musculus_ENSMUSP00000073720
Latimeria_chalumnae_ENSLACP00000021723
Xenopus_tropicalis_ENSXETP00000018837
Lepisosteus_oculatus_ENSLOCP00000014922
Danio_rerio_ENSDARP00000069635
Gasterosteus_aculeatus_ENSGACP0000001369
Oreochromis_niloticus_ENSONIP00000001723
Oryzias_latipes_ENSORLP00000016673
Takifugu_rubripes_ENSTRUP00000037164
Anolis_carolinensis_ENSACAP00000004153
Latimeria_chalumnae_ENSLACP00000009064
Lepisosteus_oculatus_ENSLOCP00000018301
Danio_rerio_ENSDARP00000145029
Takifugu_rubripes_ENSTRUP00000029002
Oreochromis_niloticus_ENSONIP00000007502
Gasterosteus_aculeatus_ENSGACP0000001269
bmp2
bmp4
bmp16
0.3

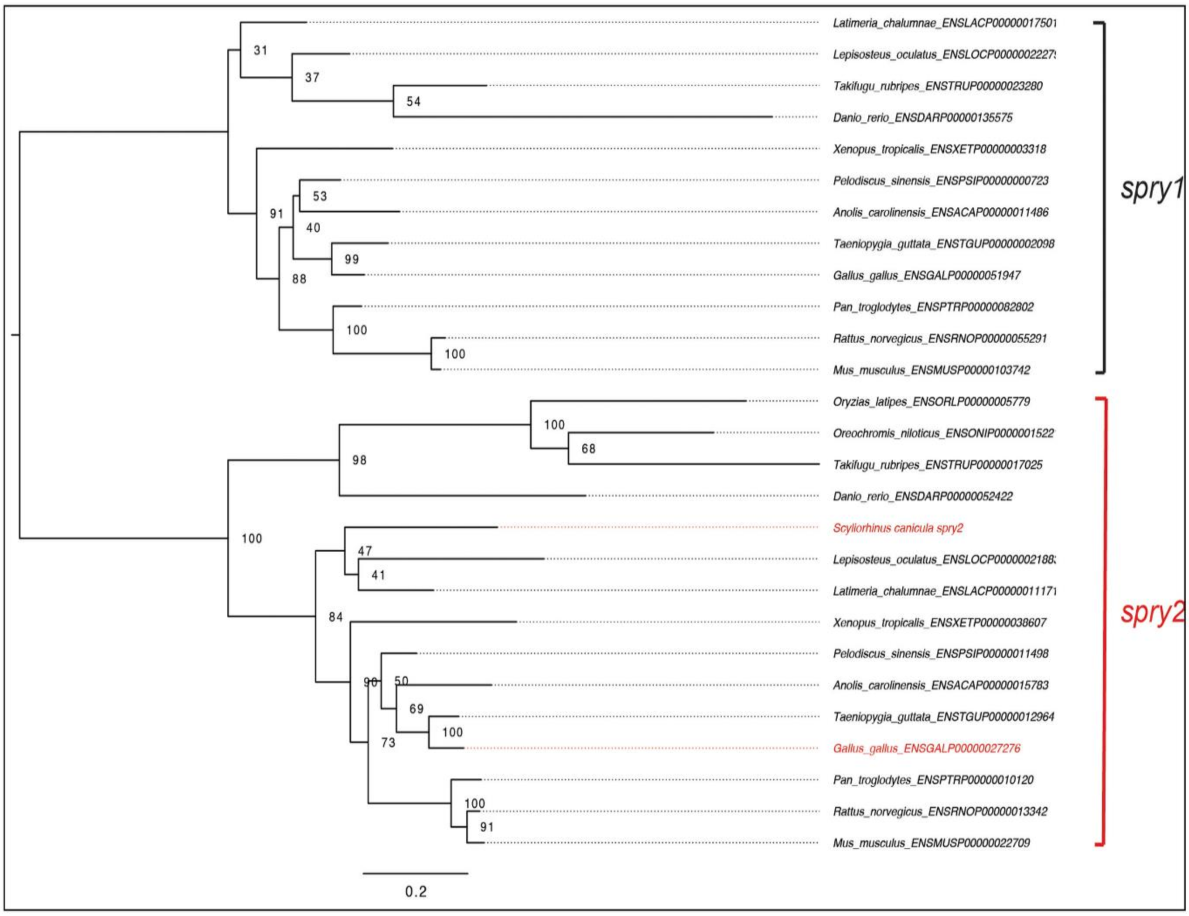

Fig. S1. Phylogenetic gene trees reconstructed from protein coding sequences extracted from www.ensembl.org. Species included in the analysis were selected based on their phylogenetic position. Ensembl sequences were aligned to *S. canicula* sequences obtained during probe synthesis steps (see methods). Sequences were aligned using MUSCLE[60]. A maximum likelihood tree was generated from 100 bootstrap replications using PHYML with a GTR substitution model[61]. Trees were edited in FigTree v1.4.3.

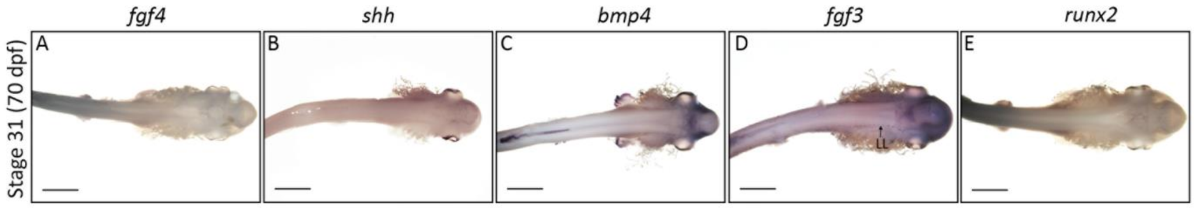

Fig. S2. Dorsal denticle placodes are not visible at stage 31 (~70 dpf). Wholemount *IS*H for *fgf4*, *shh*, *bmp4*, *fgf3* and *runx2* revealed that dorsal denticle placodes were not present at developmental Stage 31 (~70 dpf). Patterning of the lateral line sensory system (LL) is visible, expressing *fgf3*. Scale bars are 2000µm.

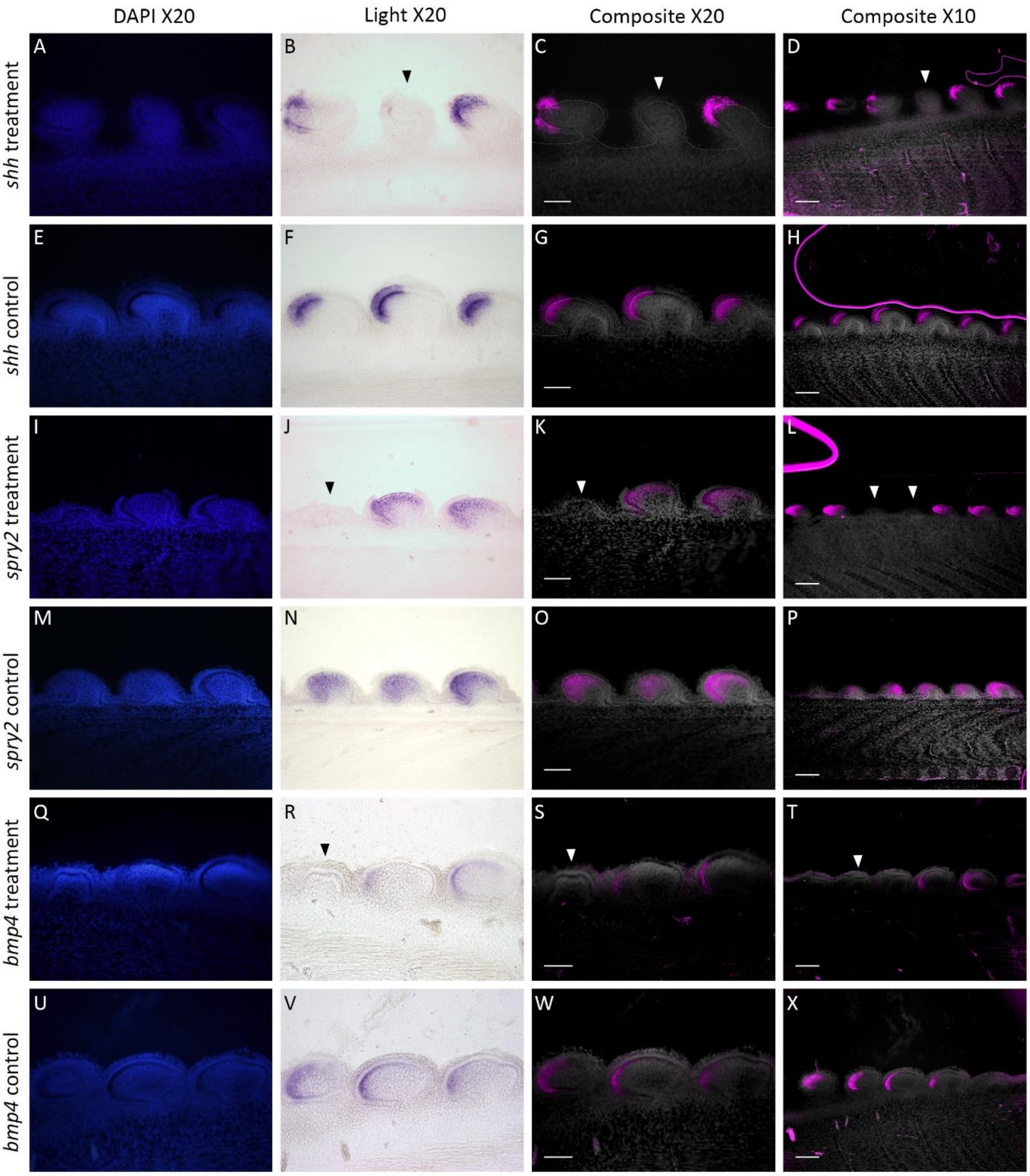

Fig. S3. Individual vibratome section images comprising false-colored ISH composite images. Vibratome sections of whole mount *IS*H are shown, for both SU5402 (treatment) and DMSO (control) beaded samples. Images are shown for both DAPI (blue) and light channels. The composite images are shown at both X10 and X20 magnification. Scale bars lengths are D, H, L, P, T and X = 100µm, and C, G, K, O, S and W = 50µm.

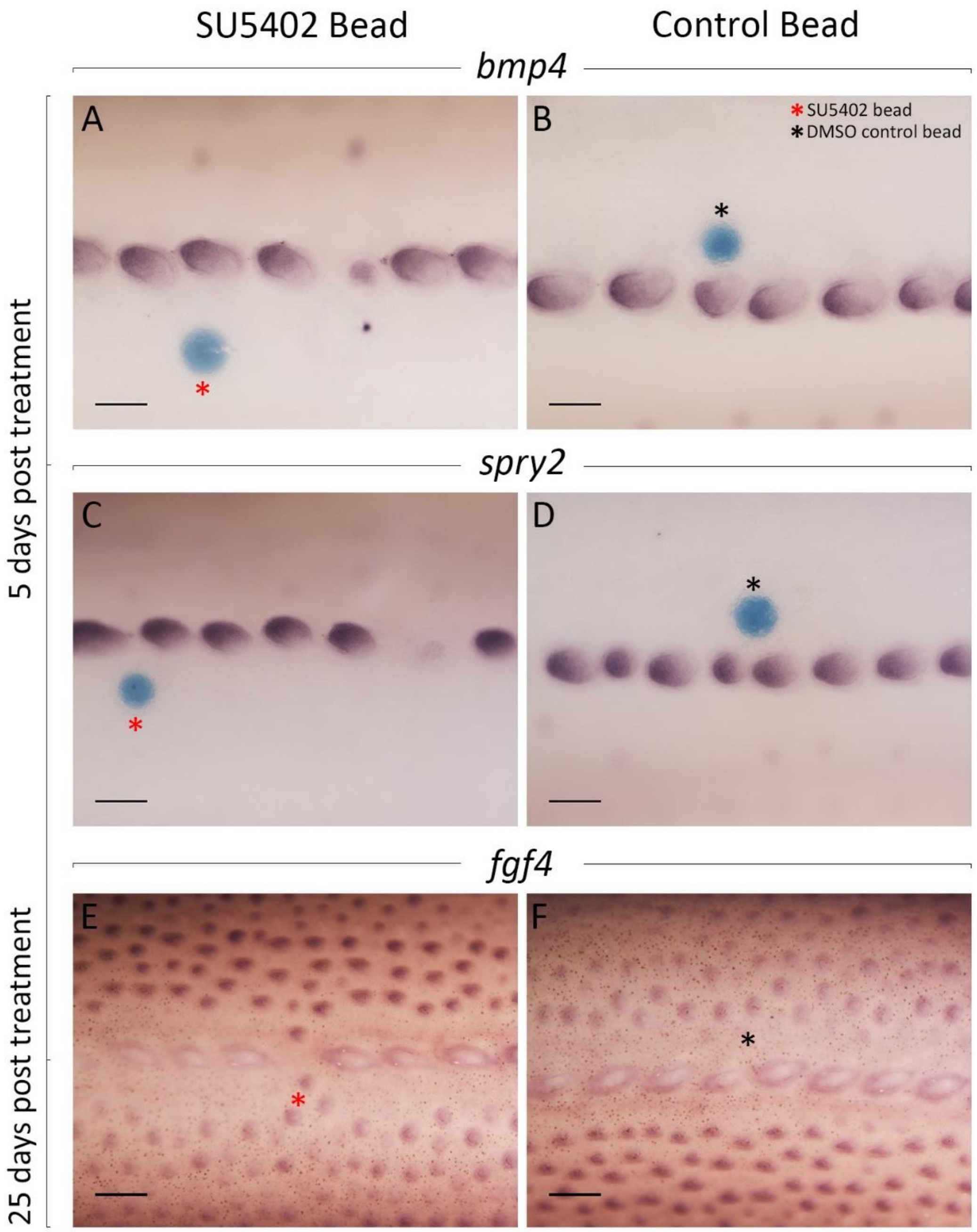

Fig. S4. Replicates of beaded shark embryos after whole-mount ISH. Whole mount *IS*H show additional shark embryo replicates after beading with SU5402 and DMSO as a control. Scale bar lengths are A-D = 200µm, and E, F = 300µm.

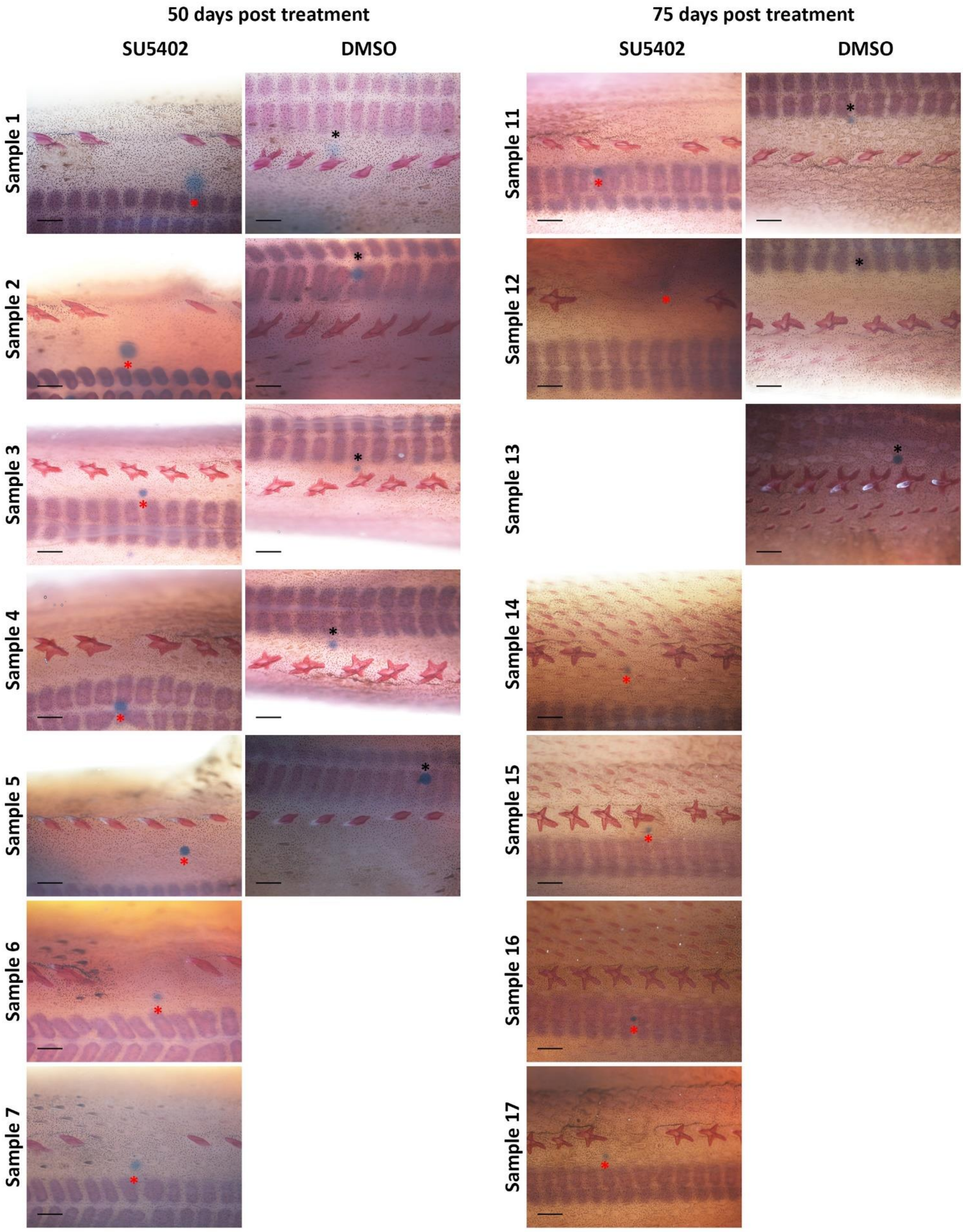

50 days post treatment
75 days post treatment
SU5402
DMSO
SU5402
DMSO
Sample 1
Sample 2
Sample 3
Sample 4
Sample 5
Sample 6
Sample 7
Sample 11
Sample 12
Sample 13
Sample 14
Sample 15
Sample 16
Sample 17

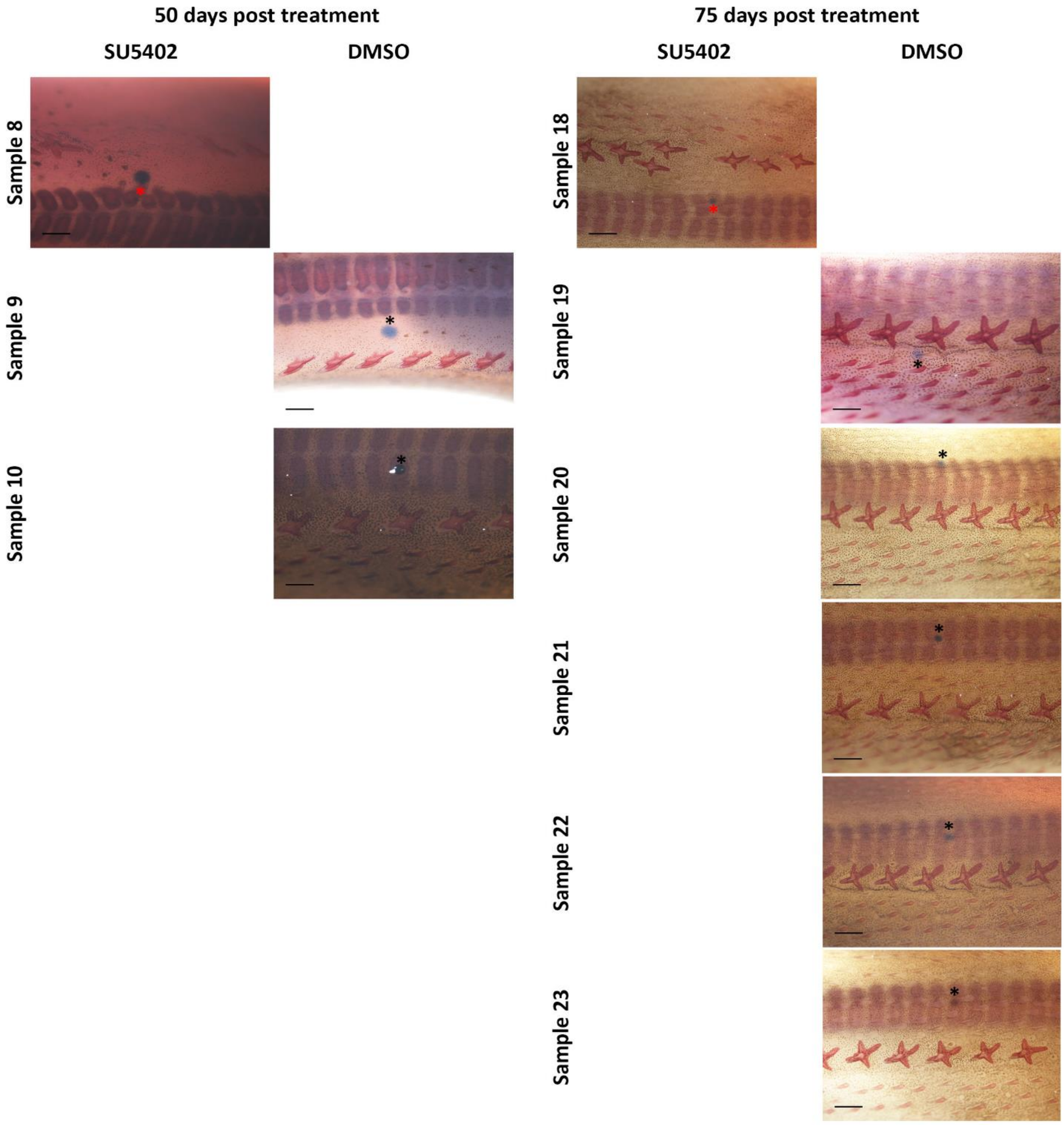

Fig. S5. Replicates of clear and stained shark embryos showing RD response to SU5402 beading. Replicates of alizarin red stained embryos treated with either SU5402 or DMSO loaded beads are shown at 50 dpt and 75 dpt. Embryos were treated with either one SU5402 bead and one DMSO bead adjacent to each dorsal row (Samples 1-5, Samples 11-12), or only one bead type per embryo (Samples 6-10, Samples 13-23). The SU5402 bead did not have an effect upon Samples 3, 5 and 16. Red asterisks mark SU5402 beads and black asterisks mark DMSO control beads. Scale bar lengths are 400µm.

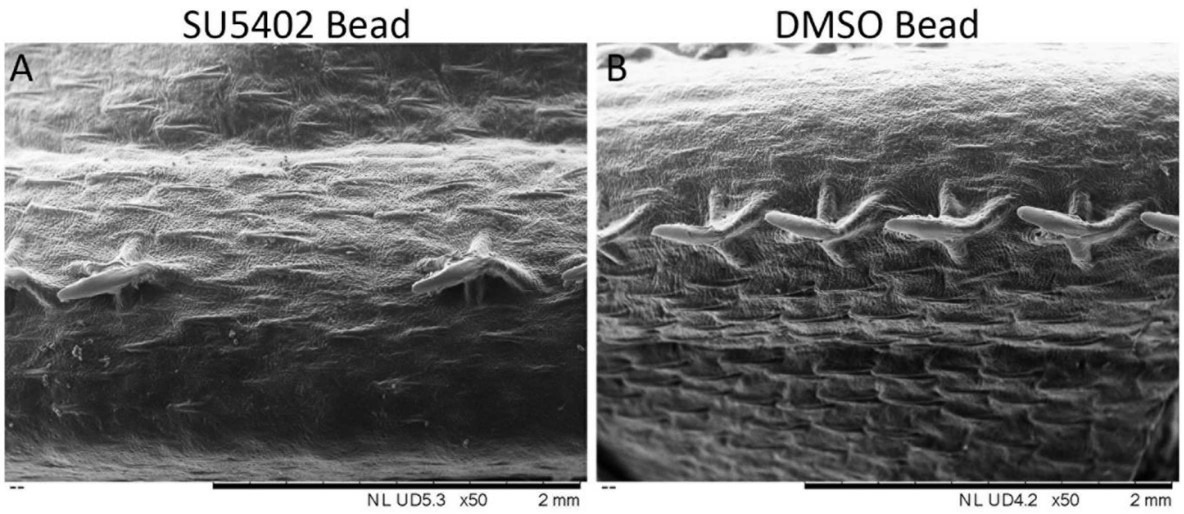

Fig. S6. SEM images of shark embryo 75 days after beading. These scanning electron microscope images show sample 14 (A) and sample 19 (B) from Supplementary Figure 5. They are 75 dpt, at developmental Stage 33 (150 dpf), and infilling of body denticles is visible in the SU5402 beaded individual (A). Tissue had to be dry when scanned, consequently resulting in distortion of the sample. Scale bar lengths are 2mm.

Table S1. Activator and inhibitor values for RD model. These are the RD model parameter values specified for patterning resembling squamation of the catshark, thornback ray and little skate (see Figure 5). Parameter values shown in red and underlined have been altered from the previous row. See 'Methods' for more information on RD modelling.

Species	***Activator values (u)***						***Inhibitor values (v)***					
	d_u	D_u	a_u	b_u	c_u	F_{max}	d_v	D_v	a_v	b_v	c_v	G_{max}
Catshark	0.03	0.02	0.08	-0.08	0.04	0.2	0.08	0.6	0.16	0.0	-0.05	0.5
Thornback ray	0.03	0.02	0.08	-0.08	0.04	0.2	0.035	0.895	0.16	0.0	-0.05	0.3
Little skate	0.03	0.02	0.08	-0.08	0.01	0.2	0.035	0.895	0.16	0.0	-0.05	0.3
